# The expression of the long *NEAT1_2* isoform is associated with human epidermal growth factor receptor 2-positive breast cancers

**DOI:** 10.1038/s41598-020-57759-4

**Published:** 2020-01-28

**Authors:** Erik Knutsen, Seyed Mohammad Lellahi, Miriam Ragle Aure, Silje Nord, Silje Fismen, Kenneth Bowitz Larsen, Marta Tellez Gabriel, Annica Hedberg, Sunniva Stordal Bjørklund, Anna Mary Bofin, Gunhild Mari Mælandsmo, Therese Sørlie, Elin Synnøve Mortensen, Maria Perander, Oslo Breast Cancer Research Consortium (OSBREAC), Jürgen Geisler, Solveig Hofvind, Tone F. Bathen, Elin Borgen, Anne-Lise Børresen-Dale, Olav Engebråten, Øystein Garred, Gry Aarum Geitvik, Anita Langerød, Bjørn Naume, Hege G. Russnes, Ellen Schlichting, Ole Christian Lingjærde, Vessela N. Kristensen, Helle Kristine Skjerven, Thomas Papathomas, Olaf-Johan Hartman-Johnsen, Kristine Kleivi Sahlberg

**Affiliations:** 1 Department of Medical Biology, Faculty of Health Sciences, UiT – The Arctic University of Norway, Tromsø, Norway; 2 Department of Cancer Genetics, Institute for Cancer Research, The Norwegian Radium Hospital, Oslo University Hospital, Oslo, Norway; 3 Department of Clinical Pathology, University Hospital of North Norway, Tromsø, Norway; 4 www.osbreac.no, Oslo, Norway; 5 Department of Clinical and Molecular Medicine, Norwegian University of Science and Technology, Trondheim, Norway; 6 Department of Tumor Biology, Institute for Cancer Research, The Norwegian Radium Hospital, Oslo University Hospital, Oslo, Norway; 7 Department of Oncology, Akershus University Hospital, Lørenskog, Norway; 8 Institute for Clinical Medicine, Faculty of Medicine, University of Oslo, Oslo, Norway; 9 Division of Medicine, Akershus University Hospital, Lørenskog, Norway; 10 Cancer Registry of Norway, Oslo, Norway; 11 Oslo and Akershus University College of Applied Sciences, Faculty of Health Science, Oslo, Norway; 12 Department of Circulation and Medical Imaging, Norwegian University of Science and Technology (NTNU), Trondheim, Norway; 13 Department of Pathology, Division of Diagnostics and Intervention, Oslo University Hospital, Oslo, Norway; 14 Department of Oncology, Division of Surgery and Cancer and Transplantation Medicine, Oslo University Hospital, Oslo, Norway; 15 Section for Breast- and Endocrine Surgery, Department of Cancer, Division of Surgery, Cancer and Transplantation Medicine, Oslo University Hospital, Oslo, Norway; 16 Centre for Cancer Biomedicine, University of Oslo, Oslo, Norway; 17 Department of Computer Science, University of Oslo, Oslo, Norway; 18 Department of Clinical Molecular Biology and Laboratory Science (EpiGen), Division of Medicine, Akershus University Hospital, Lørenskog, Norway; 19 0Breast and Endocrine Surgery, Department of Breast and Endocrine Surgery, Vestre Viken Hospital Trust, Drammen, Norway; 20 Department of Pathology, Vestre Viken Hospital Trust, Drammen, Norway; 21 Østfold Hospital, Østfold, Norway; 22 Department of Research, Vestre Viken Hospital Trust, Drammen, Norway

**Keywords:** Breast cancer, Breast cancer

## Abstract

The long non-coding RNA *NEAT1* locus is transcribed into two overlapping isoforms, *NEAT1_1* and *NEAT1_2*, of which the latter is essential for the assembly of nuclear paraspeckles. *NEAT1* is abnormally expressed in a wide variety of human cancers. Emerging evidence suggests that the two isoforms have distinct functions in gene expression regulation, and recently it was shown that *NEAT1_2*, but not *NEAT1_1*, expression predicts poor clinical outcome in cancer. Here, we report that *NEAT1_2* expression correlates with HER2-positive breast cancers and high-grade disease. We provide evidence that *NEAT1_1* and *NEAT1_2* have distinct expression pattern among different intrinsic breast cancer subtypes. Finally, we show that *NEAT1_2* expression and paraspeckle formation increase upon lactation in humans, confirming what has previously been demonstrated in mice.

## Introduction

The long non-coding RNA (lncRNA) *NEAT1* (Nuclear Paraspeckle Assembly Transcript 1) has recently gained considerable attention as it is abnormally expressed in human diseases, including cancer and neurodegenerative disorders. The *NEAT1* gene is transcribed into two isoforms, *NEAT1_1* of 3.7 kb and *NEAT1_2* of 22.3 kb, where *NEAT1_1* completely overlaps with the 5′ end of *NEAT1_2*^1^. *NEAT1_2* is essential for the assembly of paraspeckles, dynamic nuclear ribonucleoprotein complexes that phase-separate from the nucleoplasm to form liquid drop-like structures^2–7^. In contrast, *NEAT1_1* expression is not sufficient to induce paraspeckle formation, and recent reports suggest that *NEAT1_1* can localize to structures that are distinct from paraspeckles^7,8^. *NEAT1* expression and paraspeckle formation are upregulated in response to a variety of cellular stressors including mitochondrial stress, proteasome inhibition, oncogene-induced replication stress, hypoxia, heat shock, and viral infections^9–18^. It is now generally accepted that *NEAT1* and paraspeckles regulate gene expression at both transcriptional and post-transcriptional levels by acting as hubs that sequester specific gene regulatory proteins and mRNAs^16–20^. Several lines of evidence suggest that *NEAT1* and paraspeckles play critical roles in stress response pathways in general, and at specific developmental stages. *NEAT1* knockout mice display compromised mammary gland development and corpus luteum formation^21,22^. Moreover, it was recently shown that maternal and zygotic *NEAT1*-depletion frequently led to early developmental arrest at the 16- or 32-cell stage in mouse embryonic cells^23^.

Cancer cells are exposed to a variety of extrinsic and intrinsic stressors like hypoxia, proteotoxicity, DNA damage, and reactive metabolic intermediates^24^. Such malignancy-associated stress has been shown to induce *NEAT1* expression and paraspeckle formation *in vivo*^15,16^. *NEAT1* levels are elevated in hypoxic regions of breast cancer cell line xenografts, and skin tumors induced by genotoxic stress in mice, display increased *NEAT1* expression and paraspeckle formation^15,16^. In consistence with these observations, *NEAT1* is overexpressed in many cancers^15,16,25,26^. In most cases, *NEAT1* expression is associated with aggressive disease and poor clinical outcomes^15,16,25,26^.

Breast cancer is the most common type of cancer in women and covers a broad spectrum of different malignant neoplasms with clinical and genomic heterogeneity^27,28^. In clinical diagnosis, breast cancer is classified according to histological grade, Ki-67 proliferative index, and to the expression of hormone and growth factor receptors estrogen receptor (ER), progesterone receptor (PgR), and human epidermal growth factor receptor 2 (HER2). The classification of breast cancer has been stratified by gene expression profiling leading to the identification of a 50-gene signature (PAM50) that groups breast cancer into luminal A, luminal B, HER2-enriched, basal-like, and normal-like intrinsic subtypes^29–31^. Several studies have demonstrated that *NEAT1* is required for proliferation and survival of breast cancer cell lines^12,16,21,32–35^. Moreover, *NEAT1* is frequently overexpressed in breast tumor samples compared to adjacent normal tissue and is associated with poor overall survival^16,34–37^. Recently, genomic analyses of 360 primary breast tumors showed that the core promoter of the *NEAT1* gene is frequently mutated in cancer and most of these mutations are associated with loss of expression in *in vitro* assays^38^. In addition, focal deletions within the *NEAT1* gene were found in 8% of breast cancers, and mutations are frequently found in the exonic region^38,39^. This suggests that *NEAT1* expression might either protect or enhance cancer initiation and progression dependent on tumor stage. Moreover, a growing body of experimental evidence shows that the two *NEAT1* isoforms have distinct physiological functions^40,41^. Therefore, it is important to address the relative contribution of *NEAT1_1* and *NEAT1_2* in cancer progression.

In this study, we have examined the relationship between *NEAT1_2* expression and breast cancer subtypes by performing RNA-FISH analyses on core needle biopsies using probes solely recognizing the *NEAT1_2* isoform. We report that *NEAT1_2* expression associates with HER2-positive breast cancers, and with high tumor grade. This is verified by *in silico* analyses of microarray data from three independent breast cancer cohorts showing that *NEAT1_2* is most highly expressed in luminal B and HER2-enriched cancers. Interestingly, we present evidence suggesting that *NEAT1_1* expression shows a distinct distribution among breast cancer subtypes compared to *NEAT1_2*, being highest in ER-positive luminal A and luminal B cancers. This indicates that the relative expression of *NEAT1_1* versus *NEAT1_2* varies among the different breast cancer subclasses. Finally, we report that *NEAT1_2* and paraspeckle formation are induced *in vivo* in human luminal epithelial cells during lactation.

## Results

### *NEAT1_2* expression is associated with high tumor grade and HER2 positive breast cancers

**Figure 1:**
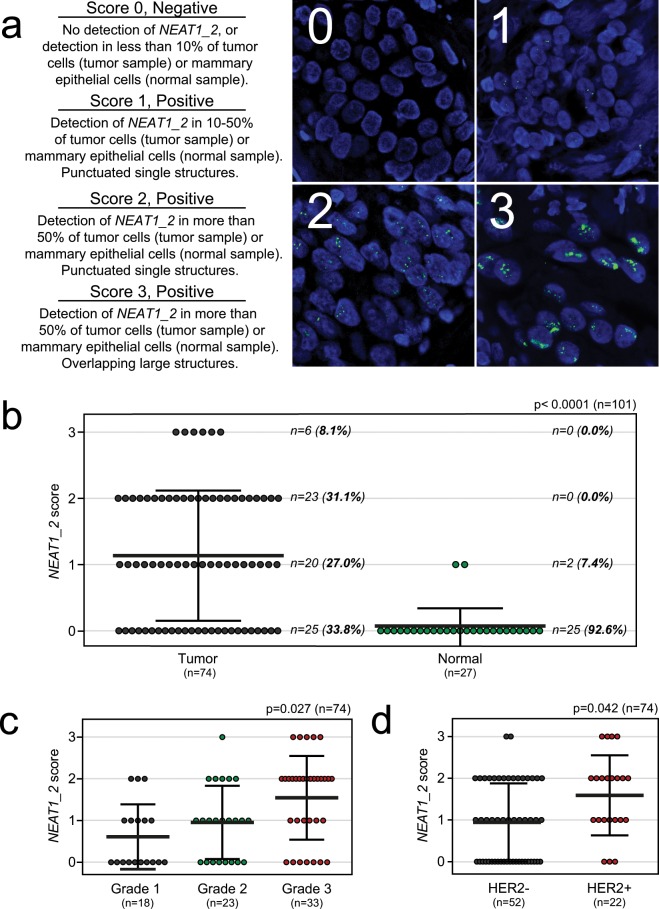
*NEAT1_2* expression and paraspeckle formation correlate with tumor grade and HER2 expression in breast cancer. (**a**) RNA-FISH analyses of *NEAT1_2* in formalin-fixed paraffin-embedded needle biopsies from breast cancer and benign samples. *NEAT1_2* expression is scored from “0” to “3” based on punctuated nuclear *NEAT1_2* signals according to the indicated criteria. For tumor samples, only tumor cells were included in the scoring, while for normal samples, only epithelial cells were included. (**b**) *NEAT1_2* is more highly expressed in tumor versus normal tissue. (**c**) *NEAT1_2* expression associates with tumor grade. (**d**) *NEAT1_2* expression associates with HER2 expression. Data are presented as mean (thick black line) ± standard deviation (thin black lines). Circles represent single patient scores. Statistical significance was calculated using the Mann Whitney test (b) or Chi square test (c) and (d). Data were considered statistically significant when p ≤ 0.05.

**Figure 2:**
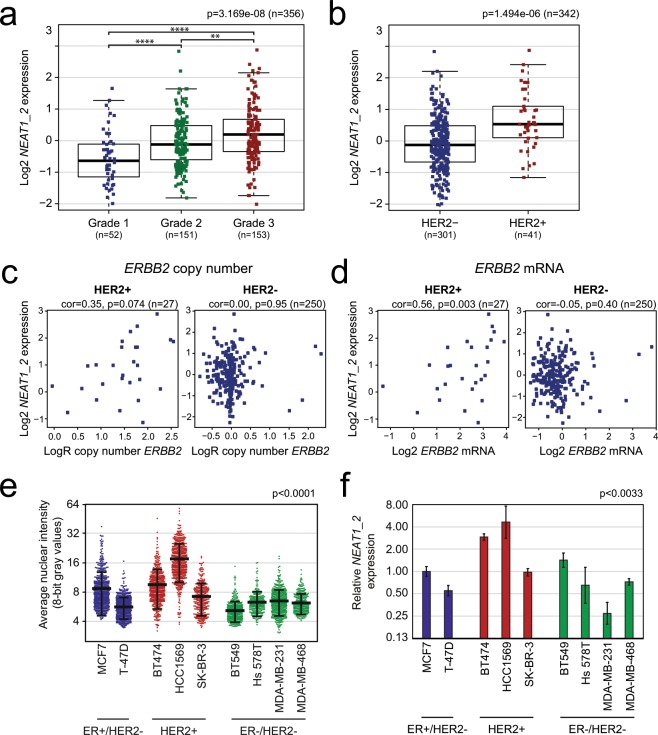
The association between *NEAT1_2* expression and HER2 status is verified in an independent breast cancer cohort and in breast cancer cell lines. (**a**) *NEAT1_2* expression was analyzed in microarray gene expression profiling data from patients in the Oslo2 cohort and correlated to tumor grade. The Kruskal-Wallis test was used to calculate whether any groups are significantly different from each other and Wilcoxon Rank-Sum test was used in post-testing for significant differences between pairs of groups. (***p ≤ 0.0001; **p ≤ 0.01). (**b**) *NEAT1_2* expression levels were correlated to HER2 status. The Wilcoxon Rank-Sum test was used to test for significant differences between the groups. (**c**) and (**d**) *NEAT1_2* expression positively correlates with *ERBB2* copy number (c) and *ERBB2* mRNA expression (d) in HER2-positive, but not in HER2-negative, patients. Correlation was calculated using Spearman’s rank correlation. (**e**) Breast cancer cell lines were subjected to *NEAT1_2* RNA-FISH and *NEAT1_2*-specific signal intensity per nucleus in at least 250 cells was quantitated. Data are given as mean (thick black line) ±standard deviation (thin black lines). Circles represent single cell intensities. The Kruskal-Wallis test was used to calculate whether any groups are significantly different from each other. (**f**) RNA was isolated from breast cancer cell lines and the expression of *NEAT1_2* was determined by RT-qPCR. The geometric means of *B2M*, *GAPDH*, and *RPLP0* were used for normalization. The mean value ± standard deviation of three biological independent experiments is presented as fold change relative to MCF7 *NEAT1_2* expression. Statistical significance was calculated using the Kruskal-Wallis test. Data were considered statistically significant when p ≤ 0.05.

The *NEAT1_2* isoform is essential for the assembly of paraspeckles that regulate the expression of specific genes at certain cellular circumstances^1,16–20^. Recently, it was shown that the expression of *NEAT1_2*, but not *NEAT1_1*, predicts progression-free survival of ovarian cancer treated with platinum-based chemotherapy^15^. This prompted us to specifically investigate the expression of *NEAT1_2* in breast cancer. To determine the relationship between breast cancer subtypes and both *NEAT1_2* expression and associated paraspeckle formation, we performed *NEAT1_2*-specifc RNA-FISH analyses on 74 formalin-fixed paraffin-embedded needle biopsies taken from females at the time of diagnosis of breast cancer. The samples were selected to represent cancers pathologically classified as luminal A (n = 23), luminal B (n = 29), triple negative/basal-like (n = 14) and HER2-positive (n = 8). We also included 27 non-cancerous breast samples in the study (23 fibroadenomas, 3 mammary reduction, and 1 *BRCA1* prophylactic mastectomy). Cancer cells were identified by experienced pathologists, and *NEAT1_2* expression was manually scored from “0” to “3” based on the presence and morphology of punctuated nuclear signals corresponding to paraspeckles (Fig. 1a). Samples scored as “1”, “2”, and “3” were defined as *NEAT1_2*-positive. Forty-nine patients (66%) were positive for *NEAT1_2* expression (Fig. 1b). In all cases, the expression was strictly restricted to cancer cells, with no detectable *NEAT1_2* signals in surrounding stromal tissue, infiltrating immune cells, or in unaffected breast tissue. In sharp contrast, only 2 out of 27 benign breast tissue samples were *NEAT1_2*-positive (7.4%), both samples being scored as “1” (Fig. 1b). Clinicopathological characteristics were acquired from each patient and correlated with *NEAT1_2* expression (Table 1). *NEAT1_2* levels significantly associated with tumor grade (Chi square test p = 0.027; Fig. 1c, Table 1), confirming what has previously been reported by others on total *NEAT1*. Interestingly, *NEAT1_2* expression also associated with HER2 positive breast cancers (Chi square test p = 0.042; Fig. 1d, Table 1). To verify these results, we analyzed microarray expression data from 381 breast cancer patients (Oslo2) using data generated by a *NEAT1_2*-specific probe^42^. We confirmed that *NEAT1_2* expression was associated with grade (Kruskal-Wallis test p = 3.169e-08; Fig. 2a) and HER2 status (Wilcoxon Rank-Sum test p = 1.49e-06; Fig. 2b). Intriguingly, we also found that *NEAT1_2* expression was significantly lower in ER-positive tumors compared to ER-negative tumors in this cohort (Wilcoxon Rank-Sum test p = 0.005155; Supplementary Fig. 1). To further determine the relationship between *NEAT1_2* and HER2 expression in the Oslo2 cohort, we investigated the correlation between *NEAT1_2* levels and *ERBB2* copy number and mRNA expression in HER2-positive and HER2-negative cancers. A close to significant positive correlation was found between *NEAT1_2* expression and *ERBB2* copy number (Spearman’s rank correlation R = 0.35, p = 0.074; Fig. 2c, left panel), and a significant positive correlation was found between *NEAT1_2* and *ERBB2* mRNA expression (Spearman’s rank correlation R = 0.56, p = 0.003; Fig. 2d, left panel). As expected, no correlation was found between *NEAT1_2* and *ERBB2* amplification or mRNA expression in HER2-negative cancers (Fig. 2c,d, right panel). Finally, we assessed the expression of *NEAT1_2* by RNA-FISH and RT-qPCR in nine breast cancer cell lines classified according to the expression of hormone- and growth factor receptors into ER/PgR-positive HER2-negative cells (MCF7, T-47D), HER2-positive cells (BT474, HCC1569, SK-BR-3), and triple negative cells (BT549, Hs 578T, MDA-MB-231, MDA-MB-468)^43^. In consistence with previous reports, the morphology, as well as the number and size of *NEAT1_2*-containing paraspeckles, varied substantially between the different cell lines (Supplementary Fig. 2)^44^. We also observed cell-to-cell variations within each cell line. In general, both the number and size of *NEAT1_2*-containing punctas were hard to determine as they frequently formed clusters. We therefore measured the average intensities of *NEAT_2* signals per cell in all cell lines (Fig. 2e). Interestingly, HER2-positive BT474 and HCC1569 clearly expressed the highest levels of *NEAT1_2*. Moreover, *NEAT1_2* expression levels in HER2-positive SK-BR-3 cells were only exceeded by those in MCF7 cells. This was confirmed by RT-qPCR analyses using primers specifically amplifying the *NEAT1_2* isoform (Fig. 2f). Generally, results obtained by imaging and RT-qPCR were concordant, only showing deviations for the BT549 cell line. We conclude that high *NEAT1_2* expression is associated with HER2-positive breast cancer and with high-grade disease. Moreover, the presence of *NEAT1_2* and paraspeckles are highly specific for cancer cells, and not present or very lowly expressed in surrounding normal cells or non-cancerous breast epithelial cells.

**Table 1:** Clinicopathological variables and *NEAT1_2* expression in breast cancer screening cohort (n = 74). The Chi square test (χ^2^-value) was used to calculate p-values.

Variable, n(%)		*NEAT1_2* expression				*p*	Total (n = 74)
		0 (n = 25)	1 (n = 20)	2 (n = 23)	3 (n = 6)		
Age at diagnosis	<55	10 (34.5)	8 (27.6)	8 (27.6)	3 (10.3)	0.920	29 (39.2)
	≥55	15 (33.3)	12 (26.7)	15 (33.3)	3 (6.7)		45 (60.8)
Histologic grade	1	10 (55.6)	5 (27.8)	3 (16.7)	0 (0.0)	0.027*	18 (24.3)
	2	8 (34.8)	9 (39.1)	5 (21.7)	1 (4.3)		23 (31.1)
	3	7 (22.2)	6 (18.2)	15 (45.5)	5 (15.2)		33 (44.6)
Tumor type	NST	20 (29.9)	20 (29.9)	22 (32.8)	5 (7.5)	0.156	67 (90.5)
	ILC	3 (100.0)	0 (0.0)	0 (0.0)	0 (0.0)		3 (4.1)
	Other invasive carcinoma^a^	2 (50.0)	0 (0.0)	1 (25.0)	1 (25.0)		4 (5.4)
Tumor diameter^**b**^	<20 mm	14 (37.8)	12 (32.4)	7 (18.9)	4 (10.8)	0.213	37 (53.6)
	≥20 mm	11 (34.4)	6 (18.8)	13 (40.6)	2 (6.3)		32 (46.4)
Lymph node metastasis^**b**^	Negative	17 (35.5)	14 (29.2)	13 (27.1)	4 (8.3)	0.990	48 (67.6)
	Positive	8 (34.8)	6 (26.1)	7 (30.4)	2 (8.7)		23 (32.4)
ER	Negative (<1%)	4 (16.7)	7 (29.2)	11 (45.8)	2 (8.3)	0.131	24 (32.4)
	Positive (≥1%)	21 (42.0)	13 (26.0)	12 (24.0)	4 (8.0)		50 (67.6)
PgR	Negative (<1%)	6 (20.7)	8 (27.6)	12 (41.4)	3 (10.3)	0.226	29 (39.2)
	Positive (≥1%)	19 (42.2)	12 (26.7)	11 (24.4)	3 (6.7)		45 (60.8)
HER2	Negative (0, 1+, 2+/no ISH amp)	22 (42.3)	13 (25.0)	15 (28.8)	2 (3.8)	0.042*	52 (70.3)
	Positive (2+/ISH amp, 3+)	3 (13.6)	7 (31.8)	8 (36.4)	4 (18.2)		22 (29.7)

^a^Tubulolobular carcinoma (n = 1), metaplastic squamous cell carcinoma (n = 1), mucinous carcinoma (n = 1), apocrine carcinoma (n = 1).

^b^Patient(s) data missing.

^*^P-value significant.

Invasive carcinoma of no special type (NST), invasive lobular carcinoma (ILC), *in situ* hybridization (ISH), amplification (amp).

### *NEAT1_2* expression is associated with the HER2-enriched and luminal B breast cancer subtypes

**Figure 3:**
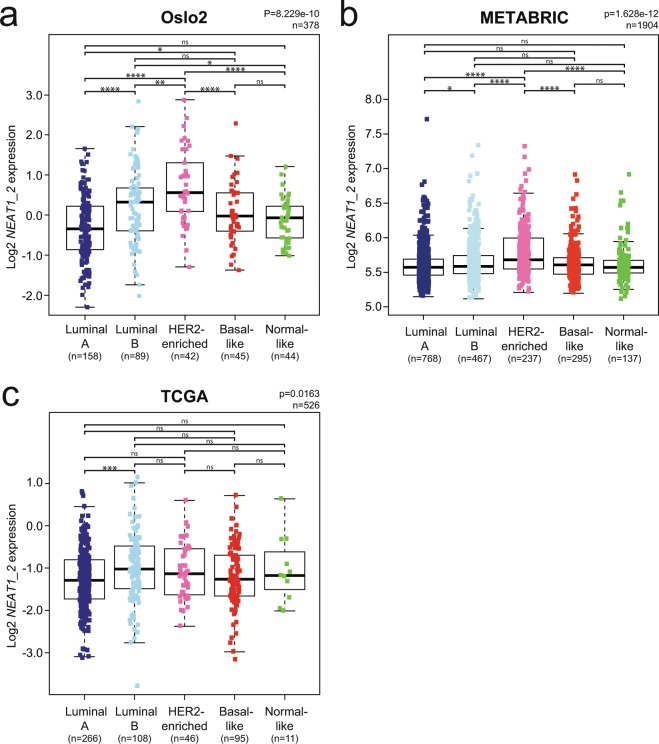
*NEAT1_2* is most highly expressed in HER2-enriched and luminal B intrinsic breast cancer subtypes in three independent cohorts. (**a**–**c**) *NEAT1_2* expression in PAM50 intrinsic breast cancer subtypes from patients of the Oslo2 (**a**), METABRIC (**b**), and TCGA (**c**) breast cancer cohorts. Subtypes were determined using the PAM50 algorithm. The Kruskal-Wallis test was used to calculate whether any groups are significantly different from each other and Wilcoxon Rank-Sum test was used in post-testing for significant differences between pairs of groups. (****p ≤ 0.0001; ***p ≤ 0.001; **p ≤ 0.01; *p ≤ 0.05; ns, p > 0.05).

We demonstrated above that *NEAT1_2* expression associates with HER2-positive breast cancer. HER2 overexpressing cancers are in most cases classified as HER2-enriched or luminal B using the PAM50 gene expression signature identifier. To assess the association between *NEAT1_2* expression and intrinsic breast cancer subtypes, we analyzed microarray gene expression data derived from the Oslo2 cohort described above, and two publicly available breast cancer patient cohorts, METABRIC^28^ and The Cancer Genome Atlas (TCGA)^45^. Patients were subtyped using the PAM50 algorithm^31^, and only data generated from probes solely recognizing the *NEAT1_2* isoform were considered. In all three cohorts, *NEAT1_2* was most highly expressed in breast cancers classified as HER2-enriched and luminal B, but with different intrinsic distributions (HER2-enriched > luminal B in Oslo2 and METABRIC; luminal B > HER2-enriched in TCGA) (Fig. 3a–c). Luminal A breast cancers had the lowest expression of *NEAT1_2* in all three cohorts. Taken together, these results are in accordance with the observed correlation between *NEAT1_2* expression and HER2-status.

### *NEAT1_1* expression is highest in luminal A and luminal B breast cancers

**Figure 4:**
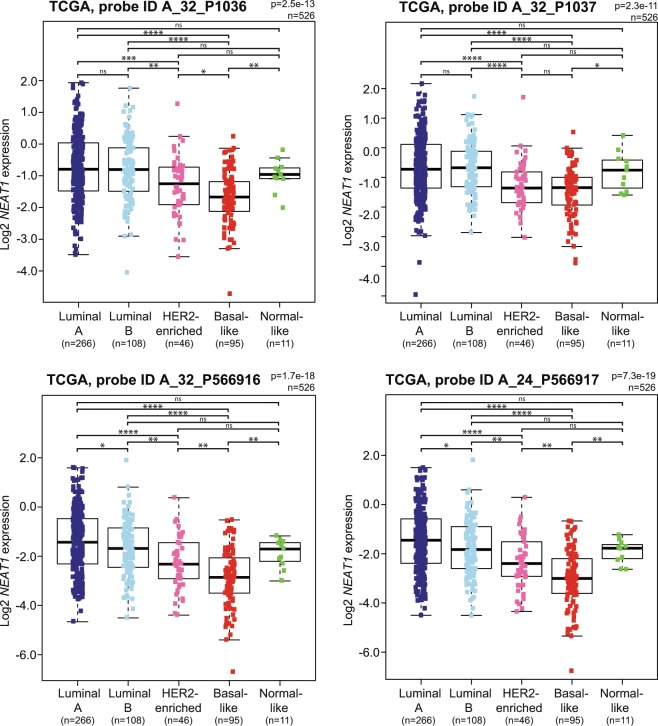
Total *NEAT1* is most highly expressed in the luminal subtypes in the TCGA breast cancer cohort. Expression of total *NEAT1* in PAM50 intrinsic breast cancer subtypes was determined using data generated from four independent microarray probes in the TCGA cohort. The Kruskal-Wallis test was used to calculate whether any groups are significantly different from each other and Wilcoxon Rank-Sum test was used in post-testing for significant differences between pairs of groups. (****p ≤ 0.0001; ***p ≤ 0.001; **p ≤ 0.01; *p ≤ 0.05; ns, p > 0.05).

**Figure 5:**
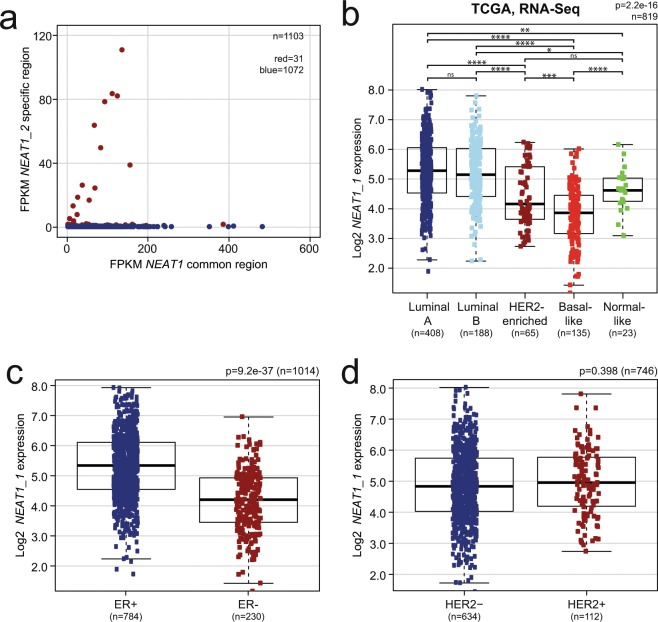
*NEAT1_1* is most highly expressed in the luminal breast cancer subtypes in the TCGA RNA-Seq cohort. (**a**) *NEAT1* common region FPKM (Fragments Per Kilobase Million) is plotted against *NEAT1_2* specific-region FPKM for each patient. Each dot represents one patient. Samples with more than 1 FPKM for the *NEAT1_2-*specific region (red dots) were excluded from further analysis. (**b**) *NEAT1_1* is most highly expressed in luminal A and luminal B tumors. The Kruskal-Wallis test was used to calculate whether any groups are significantly different from each other and Wilcoxon Rank-Sum test was used in post-testing for significant differences between pairs of groups. (**c**) *NEAT1_1* expression associates with ER expression status. (**d**) *NEAT1_1* expression does not associate with HER2 status. Statistical significance was calculated in (**c**) and (**d**) using Wilcoxon Rank-Sum test. Data were considered statistically significant when p ≤ 0.05. (****p ≤ 0.0001; ***p ≤ 0.001; **p ≤ 0.01; *p ≤ 0.05; ns, p > 0.05).

Previous reports have demonstrated that the *NEAT1* gene is transcriptionally activated by ERα in both prostate and breast cancer, and the transcript participates in a gene repressor complex that induces epithelial-mesenchymal transition (EMT) in a mouse model of ER-positive breast cancer^25,36^. Here, we have found that the expression of the long *NEAT1_2* isoform is lower in ER-positive compared to ER-negative tumors in the Oslo2 breast cancer cohort (Supplementary Fig. 1). This potential discrepancy made us analyze the expression of total *NEAT1* using microarray data derived from probes binding to both *NEAT1_1* and *NEAT1_2* from the TCGA cohort. Interestingly, total *NEAT1* expression showed a different distribution among the PAM50 subtypes compared to *NEAT1_2*, being most highly expressed in luminal A, luminal B, and normal-like cancers (Fig. 4). *NEAT1_1* is, as opposed to *NEAT1_2*, a polyadenylated transcript. To more specifically investigate the expression of *NEAT1_1* in breast cancer, we analyzed polyA-selected RNA-sequencing data from the TCGA breast cancer cohort. We only extracted data from samples that hardly displayed any mapping of fragments to the unique *NEAT1_2* region (<1.0 FPKM (Fragments Per Kilobase Million)) (Fig. 5a). Patients were then subtyped according to the PAM50 classifier, and ER and HER2 status were extracted. *NEAT1_1* showed a similar distribution among the PAM50 subtypes as total *NEAT1*, being highest in luminal A and luminal B breast cancers (Fig. 5b). In line with this, *NEAT1_1* expression clearly associated with ER-positive tumors (Wilcoxon Rank-Sum test p = 9.2e-37; Fig. 5c). Finally, we found no association between *NEAT1_1* and HER2 expression (Wilcoxon Rank-Sum test p = 0.398; Fig. 5d). To conclude, our data clearly indicate that the relative expression of *NEAT1_1* versus *NEAT1_2* varies among different breast cancer subclasses.

### *NEAT1_2* expression is upregulated in human breast tissue during lactation

**Figure 6:**
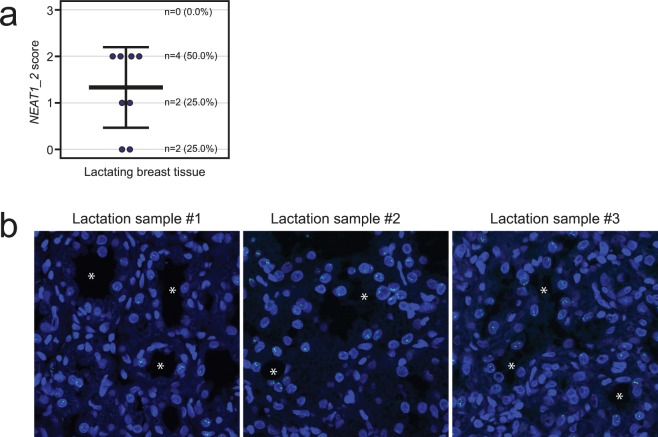
*NEAT1_2* is expressed in lactating breast tissue. **(a)** RNA-FISH analyses of *NEAT1_2* in breast tissue from lactating females (n = 8). *NEAT1_2* expression is scored from “0” to “3” based on punctuated nuclear *NEAT1_2* signals according to the indicated criteria in Fig. 1a. Data are shown as mean (thick black line) ±standard deviation (thin black lines). Circles represent single patient scores. (**b**) RNA-FISH images from three lactating females. *NEAT1_2* is visualized in green, and DAPI (blue) was used to stain the nuclei. White asterisks visualize some of the lumens within the mammary glands.

We have demonstrated that *NEAT1_2* is rarely expressed in normal human breast tissue. *NEAT1* female knock-out mice display compromised mammary gland development during puberty and pregnancy, and fail to lactate due to impaired proliferation of luminal alveolar cells^22^. This suggests that *NEAT1* has an important function in mammary gland development, and during pregnancy and lactation. In order to investigate if *NEAT1_2* is expressed during lactation in humans, we analyzed eight needle biopsies taken from females with lactation-related benign changes in the mammary gland. Importantly, 75% (n = 6) of the lactating breast tissue samples were positive for *NEAT1_2* using the same scoring scheme as above (Figs. 1a and 6a). Of note, we also had access to one sample from a pregnant woman, which was scored as *NEAT1_2* positive (score 2). In both the lactating tissue and the breast tissue from the pregnant female, the expression of *NEAT1_2* was restricted to the luminal breast epithelial cells (Fig. 6b).

## Discussion

The lncRNA *NEAT1* locus is conserved in mammalian species and encodes two overlapping transcripts, *NEAT1_1* and *NEAT1_2*, of which the latter is essential for the assembly of paraspeckles^1^. Early analyses in mice indicated that whereas *NEAT1_1* is ubiquitously expressed, the expression pattern of *NEAT1_2*, and thus the presence of paraspeckles, is more restricted^5^. Emerging evidence now suggests that *NEAT1_2* and paraspeckles play critical roles in orchestrating specific gene expression upon cellular stress and at specific developmental stages^9–18^. Importantly, it was recently shown that the expression of *NEAT1_2*, but not total *NEAT1*, was associated with aggressive cancers^15^. Here, we have specifically analyzed the expression of *NEAT1_2* in breast cancer. By performing RNA-FISH on 74 breast cancer needle biopsies, we found that *NEAT1_2* expression and paraspeckle formation associated with HER2-positive cancers. We verified this by inspecting microarray data generated by a *NEAT1_2*-specific probe from a cohort of 381 patients. Moreover, we found that *NEAT1_2* is highly expressed in HER2-positive compared to HER2-negative breast cancer cell lines. Finally, in three different breast cancer cohorts, *NEAT1_2* expression associated with HER2-enriched and luminal B PAM50 intrinsic subtypes.

Around 15–20% of all breast cancers overexpress the HER2 receptor, many of them due to the amplification of the *ERBB2* gene on chromosome 17, and HER2-driven cancers are generally aggressive^46,47^. The HER2 receptor is an orphan member of the epidermal growth factor receptor family that upon overexpression forms homodimers or heterodimers with either EGFR, HER3, or HER4, which elicit signaling pathways, including the MEK-ERK and PI3-kinase-Akt pathways, that drive tumorigenesis^46,47^. *NEAT1* expression is generally regulated at the transcriptional level, and it is reasonable to assume that HER2-signaling leads to the activation of the *NEAT1* promoter. Indeed, *NEAT1* transcription is activated by a series of stress-induced transcription factors including HIF2α, HSF1, and NF-κB, which have been shown to be constitutively upregulated or activated in HER2 overexpressing cells^48–51^. Importantly, *NEAT1* is also a p53 target gene, and oncogenic stress has been shown to upregulate *NEAT1* expression in a p53-dependent manner^15,52,53^. This might account for the relatively high expression of *NEAT1_2* observed in the wild-type p53 cell line MCF7 (Fig. 2e,f). Recently, it was suggested that high *NEAT1* expression is associated with good prognosis in p53 wild-type breast cancers^54^. However, p53 is frequently mutated in HER2-positive cancers^45^, and HCC1569 cells, which express the largest amount of *NEAT1_2* among the cell lines included in this study, carry a p53 nonsense mutation^55^. The relationship between p53 mutational status, HER2, and *NEAT1* expression in breast cancer should be a subject of future research.

As *NEAT1_1* and *NEAT1_2* are transcribed from the same promoter, it is logical to hypothesize that the expression pattern of *NEAT1_1* mirrors that of *NEAT1_2*. Importantly, by analyzing microarray data derived from probes binding to both *NEAT1* isoforms and polyA-enriched RNA-sequencing data, we found that *NEAT1_1* expression showed a different distribution among the PAM50 subtypes compared to *NEAT1_2*. Whereas *NEAT1_2* is most highly expressed in HER2-enriched and luminal B cancers, *NEAT1_1* expression is highest in ER-positive luminal A and luminal B cancers. Thus, our analyses strongly suggest that the relative levels of *NEAT1_1* versus *NEAT1_2* vary in different breast cancer subtypes. Previous reports have shown that *NEAT1* is transcriptionally activated by ERα in both prostate and breast cancer cell lines^25,36^. Recently, Li *et al*. found that *NEAT1* participates in a transcriptional repressor complex with FOXN3 and SIN3A in ER-positive breast cancer cells^36^. The complex induces EMT *in vitro* by downregulating GATA3 expression and promotes metastasis in mouse models of ER-positive breast cancer. The FOXN3-*NEAT1*-SIN3A complex also binds to and represses the promoter of the *ESR1* gene indicating the presence of a negative feed-back regulatory mechanism. Importantly, the authors suggest that the FOXN3-*NEAT1*-SIN3A complex functions independently of paraspeckles and that it is the *NEAT1_1* isoform that participates in this complex. In line with this, Chakravarty *et al*. demonstrated that *NEAT1_1*, but not *NEAT1_2*, binds directly to histone H3 and recruits ERα to the *PSMA* promoter in prostate cancer cell lines^25^. We hypothesize that in ER-positive cancers, *NEAT1_1* contributes to the tumorigenic phenotype by directly participating in transcriptional regulation at the chromatin level. This mechanism might be less important in HER2-positive cancers where increased *NEAT1_2* levels and paraspeckle formation are required for their adaptation to malignancy-associated stress and survival. A recent study of pan-cancer tissue microarrays, indeed showed that 65% of human carcinomas displayed increased number of paraspeckles compared to non-malignant tissue^15^. More importantly, the same authors reported that the expression of *NEAT1_2*, but not total *NEAT1*, predicted progression-free survival of ovarian cancer treated with platinum-based chemotherapy. *NEAT1_2* is produced when the polyadenylation signal required for the formation of *NEAT1_1*, is suppressed by a hnRNPK-dependent mechanism^7,56^. Moreover, key paraspeckle-associated proteins including NONO and SFPQ bind to and stabilize *NEAT1_2*^6^. Further experiments should be undertaken to determine their expression and subcellular localization in HER2-positive breast cancers, as well as in other cancers.

We have found that *NEAT1_2* is not expressed in normal tissue surrounding breast cancer cells at levels that can be detected by RNA-FISH. Furthermore, only 7.4% of benign breast tissue samples were *NEAT1_2* positive. Murine *Neat1* is critical for normal development of the mammary gland, and *Neat1_2* and paraspeckles were detected in 30–50% of K8/K18-positive luminal cells in adult mice^22^. The number of *Neat1_2* positive cells increased upon pregnancy and lactation. To further inspect *NEAT1* expression pattern in human mammary gland development, we performed RNA-FISH on 8 benign breast tissue samples taken from lactating women. We detected *NEAT1_2* and paraspeckles in 6 samples (75%). Our data strongly supports the observations done in mice and suggests that *NEAT1_2* and paraspeckle formation are upregulated during lactation also in humans. However, it remains to be determined at which stage in pregnancy *NEAT1_2* is upregulated. As *Neat1* knockout mice display compromised proliferation of luminal alveolar epithelial cells during pregnancy^22^, it is reasonable to hypothesize that elevated *NEAT1_2* levels are required for pregnancy-induced expansion of the epithelial compartment in humans as well. Alternatively, *NEAT1_2* might be upregulated during the differentiation of luminal alveolar cells into milk-secreting cells. The epithelium of the adult mammary gland is interspersed with mammary stem cells and progenitor cells^57^. In the future, experiments should be undertaken to determine the expression status of *NEAT1* in these cells, as this would shed light on the function of *NEAT1* in both postnatal mammary gland development and breast cancer.

We provide evidence that *NEAT1_2* expression associates with HER2-positive cancers and suggest that the relative expression of *NEAT1_1* versus *NEAT1_2* varies in breast cancer subtypes. The overlapping nature of the *NEAT1_1* and *NEAT1_2* hampers isoform-specific analyses and might affect the interpretation of expression data. *NEAT1_2* is not polyadenylated, which needs to be taken into account when analyzing polyA-enriched RNA-sequencing data. It should also be noted that RNA stability is a technical challenge when analyzing *NEAT1_2* expression in formalin fixed paraffin embedded patient samples by RNA-FISH. Nevertheless, both *NEAT1_1* and *NEAT1_2* are likely to contribute to breast cancer tumorigenesis, but through different mechanisms. The highly tumor-specific expression of *NEAT1_2* in breast cancer, makes it a promising target for future therapeutic intervention.

## Methods

### Cell culture

BT474 (ATCC® HTB-20™), BT549 (ATCC® HTB-122™), HCC1569 (ATCC® CRL-2330™), Hs 578T (ATCC® HTB-126™), MDA-MB-231 (ATCC® HTB-26™), MDA-MB-468 (ATCC® HTB-132™), MCF7 (ATCC® HTB-22™), SK-BR-3 (ATCC® HTB-30™), and T-47D (ATCC® HTB-133™) cells were all purchased from the American Type Culture Collection (ATCC). BT474, BT549, HCC1569, MDA-MB-231, MDA-MB-468, SK-BR-3, and T-47D cells were cultured in RPMI 1640 (Sigma-Aldrich) supplemented with 10% Fetal bovine serum (FBS) (Biochrom) and 1% penicillin-streptomycin (Sigma-Aldrich). BT549 cells were grown in the presence of 1.0 μg/ml insulin (Sigma-Aldrich) and T-47D cells were grown in the presence of 6.0 μg/ml insulin. Hs 578 T cells were cultured in Dulbecco’s Modified Eagle’s Medium (DMEM; Sigma-Aldrich) supplemented with 10% FBS, 1% penicillin-streptomycin, and 10.0 μg/ml insulin. MCF7 cells were cultured in Minimum Essential Medium Eagle (MEM; Sigma-Aldrich) supplemented with 10% FBS, 1% penicillin-streptomycin, and 10.0 μg/ml insulin. All cell lines were incubated in a 5% CO2 humidified incubator at 37 °C.

### RNA isolation, cDNA synthesis, and RT-qPCR

Cells were lysed in 300 µl Tri Reagent (Zymo Research) and heated for 10 min at 55 °C in order to prevent *NEAT1* from being trapped in the protein phase during isolation^44^. Total RNA was isolated with Direct-zol RNA MiniPrep (Zymo Research) according to the manufacturer’s recommendation. RNA concentration was measured by NanoDrop 2000 (Thermo Fisher Scientific). cDNA synthesis of total RNA was performed with SuperScript™ IV Reverse Transcriptase (ThermoFisher Scientific). 2.5 μM of random hexamer primer (ThermoFisher Scientific) and approximately 400 ng of template were used for the reaction. Total RNA was denaturated at 65 °C for 5 min, and cDNA was synthesized at 50 °C for 10 min.

For RT-qPCR, cDNA was mixed with FastStart Essential DNA Green Master (Roche Life Science) and 0.25 μM forward and reverse primer. All primer sequences are provided in Supplementary Table 1. The LightCycler® 96 was used for quantification, and the ΔΔCq-method was used to calculate fold change using the geometric mean of *GAPDH*, *B2M*, and *RPLPO* as internal reference.

### RNA-FISH of cells and FFPE tissue

Stellaris® *NEAT1* RNA FISH probes recognizing the *NEAT1_2* isoform (SMF-2037-1 conjugated with Quasar® 670) was purchased from LGC Biosearch Technologies. Preparation of cells and FFPE sections, hybridization, and mounting were performed according to the Stellaris® RNA FISH Probes manuals. In brief, cells were seeded onto circular coverslips in 12-well dishes and allowed to attach for 2–3 days. The cells were fixed with 4% formaldehyde and permeabilized with 70% EtOH. Hybridization was done at 37 °C in a humidifying chamber for at least 4 hours. FFPE tissue sections were cut fresh and placed at 60 °C for 45 min before being deparaffinized with xylene. Here, hybridization was performed overnight. Vectashield® Mounting Medium containing DAPI was used for mounting of both cells and FFPE sections. Images were generated using a Zeiss LSM780 confocal microscope. For cells, 3-dimensial Z-stack images were taken at 40x magnification (seven pictures, with 0.6 μm distance between each picture). Images of FFPE sections were taken at 20x magnification with no Z-stacking. All images were processed using ZEN 2012 (black edition) v8.0. *NEAT1_2* fluorescence was quantified from maximum intensity projections of confocal z-stacks using Fiji^58^ running ImageJ^59^ version 1.52n. An automatic threshold was set in the DAPI channel in order to segment individual nuclei using the wand tool. In some cases, nuclear outlines were manually traced. The average intensity in the *NEAT1_2* channel was then measured for each nucleus.

### Clinical samples

Archived FFPE needle biopsies were obtained from the Department of Pathology, University Hospital of North Norway (UNN) with corresponding hematoxylin and eosin (HE) slides from all patients. Samples from 74 patients diagnosed with breast cancer (2012–2018, age range 31–84 years), 27 normal samples (2013–2015, age range 18–68 years), 8 samples from lactating females (2013–2015, age range 25–42), and 1 sample from a pregnant female (2013, age 32) were included in the study. Approval for this study, including dispensation from the requirement of patient consent, was granted by the Norwegian Regional Committee for Medical and Health Research Ethics, approval number 2014/317. We confirm that all experiments were performed in accordance with relevant guidelines and regulations. Histological tumor grade was assessed by the Nottingham Grading System^60^. The samples were classified by pathologists as luminal A (ER+ and/or PgR+, HER2- Ki-67 < 15%), luminal B (ER+ and/or PgR+, HER2- Ki-67 ≥ 15% or ER+ and/or PgR+, HER2+), triple negative/basal-like (ER−, PgR−, HER2−), or HER2-positive (ER−, PgR−, HER2+). The cut off values for ER and PgR were 1%. Tumors with HER2 protein overexpression (IHC 3+) or with *in situ* hybridization (ISH)-detected amplified HER2 gene (IHC 2+/ISH HER2 gene amplification) were considered to be HER2 positive. *NEAT1_2* expression and clinicopathological characteristics were analyzed by the Chi square test (χ^2^-value) using SPSS version 25 (SPSS Inc., Chicago, IL, USA). P-values ≤ 0.05 (two-tailed) were considered statistically significant.

### Gene expression analyses in breast cancer cohorts

*NEAT1* gene expression was assessed in three independent breast cancer cohorts; Oslo2^42^, METABRIC^28^, and TCGA^45^. Oslo2 is a multicentre study of breast cancer patients with primary operable breast cancers enrolled from hospitals in the Oslo (Norway) region (approved by the Norwegian Regional Committee for Medical and Health Research Ethics, approval number 2016/433 and 429-04148)^42^. Gene expression data (GEO accession number GSE80999) were obtained using SurePrint G3 Human GE 8 × 60 K microarrays (Agilent Technologies, Santa Clara, CA, USA) and data were log2-transformed, quantile-normalized, and hospital-adjusted. Probe A_33_P3263538 covered part of the unique 3′ end of *NEAT1_2*. *ERBB2* mRNA expression values were derived from mRNA probes using the median value for the two probes matching this gene symbol (A_23_P89249 and A_33_P3292596). For HER2 copy number analysis, logR values (the log2-transformed value of the normalized intensity of the SNP) were extracted from raw CEL-files from Genome-Wide Human SNP Array 6.0 (Affymetrix, Santa Clara, CA, USA) using Affymetrix power tools. Segmented copy number values were generated and non-aberrant cell fraction and ploidy was calculated using the Allele-Specific Copy number Analysis of Tumors (ASCAT) package^61^. Segmented copy number data (adjusted for non-aberrant cell admixture and ploidy) were log2-transformed and made probe-centric based on the *ERBB2* mRNA expression array probe location. The METABRIC cohort is composed of 1980 breast cancer patients collected at five different hospitals in the UK and Canada^28^. Gene expression was assessed using the Illumina HT-12 v3 microarray and downloaded from the European Genome-phenome Archive (EGA) data portal. The data were log2-transformed. Probe ILMN_1675354 covered part of the unique 3′ end of *NEAT1_2*. Gene expression levels for the Caucasian fraction of the TCGA cohort (n = 526) were assayed by Agilent 244 K Custom Gene Expression G4502A-07-3^45^. The data were log2-transformed after normalization. The probe A_32_P206561 covered parts of the unique 3′ end of *NEAT1_2*, while probes A_32_P1036, A_32_P1037, A_24_P566917, and A_24_P566916 covered parts of the common region between *NEAT1_1* and *NEAT1_2*. For analysis of RNA-Seq data from TCGA, bam files aligned to GRCh38 were downloaded from https://gdc.cancer.gov/. Reads mapping to the *NEAT1* common region (Chr11: 65422798-65426532) and the *NEAT1_2* specific region (Chr11: 65426533-65445540) were counted using the featureCounts function of the Subread package specified with the –p flag (version 1.6.1)^62^. Fragments spanning the end of the *NEAT1* common region and the start of the *NEAT1_2* specific region were excluded. FPKM was calculated as [RMg * 10^9^]/[RMt * L], where RMg are reads mapping to each transcript, L is length of transcripts in bp, and RMt are total number of mapped reads. Samples with *NEAT1_2* specific region FPKM ≥ 1.0 were filtered out, leaving 1065 tumor samples for clinical analysis.

### Statistical analysis

GraphPad software version 25 (SPSS Inc., Chicago, IL, USA) was used to analyze the screening cohort (74 breast cancer needle biopsies). Analysis of significance in expression in normal versus tumor tissue was calculated using the Mann Whitney test. For analyses of clinicopathological variables and *NEAT1_2* expression the Chi square test (χ^2^-value) were used. Data were considered statistically significant when p ≤ 0.05.

For microarray and RNA-Seq expression analyses, statistical analyses were performed in R^63^. *NEAT1_2, NEAT1_1*, and *NEAT1* expression across PAM50 intrinsic subtypes and tumor grade were compared using the non-parametric Kruskal-Wallis test. For HER2 and ER expression comparison between two groups, Wilcoxon Rank-Sum test were used. Post tests between subtypes and grade were done with Wilcoxon Rank-Sum test. Spearman’s rank correlation was used for analysis of *ERBB2* copy number and mRNA expression correlation with *NEAT1_2* expression. Data were considered statistically significant when p ≤ 0.05.

## Supplementary information

Supplementary information.
