# Supplementary material for: The expression of the long NEAT1_2 isoform is associated with human epidermal growth factor receptor 2-positive breast cancers
Source: Sci Rep. 2020 Jan 28;10:1277. doi: 10.1038/s41598-020-57759-4 (PMC6987222; doi:10.1038/s41598-020-57759-4)
Supplement: Supplementary file 1 — Supplementary information. [file 41598_2020_57759_MOESM1_ESM.pdf]

The expression of the long *NEAT1_2* isoform is associated with human epidermal growth factor receptor 2-positive breast cancers

Erik Knutsen[1], Seyed Mohammad Lellahi[1], Miriam Ragle Aure[2], Silje Nord[2], Silje Fismen[3,23], Kenneth Bowitz Larsen[1], Marta Tellez Gabriel[1], Annica Hedberg[1], Sunniva Stordal Bjørklund[2], Oslo Breast Cancer Research Consortium (OSBREAC)[4†],Anna Mary Bofin[5], Gunhild Mari Mælandsmo[6], Therese Sørlie[2], Elin Synnøve Mortensen[1,3] & Maria Perander[1*].

Supplementary information:

- Supplementary figure 1
- Supplementary figure 2
- Supplementary table 1

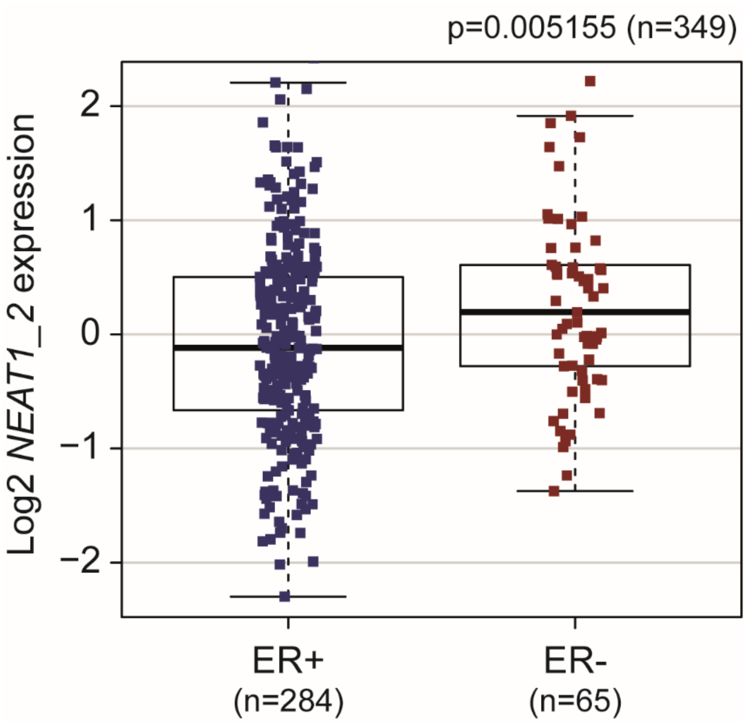

Supplementary figure 1: *NEAT1_2* expression is significantly lower in estrogen receptor (ER) positive compared to ER negative tumors. *NEAT1_2*-specific expression was analyzed in microarray expression data from breast cancer patients of the Oslo2 cohort. P-value was calculated using the Wilcoxon Rank-Sum test. Data were considered statistically significant when $p \leq 0.05$.

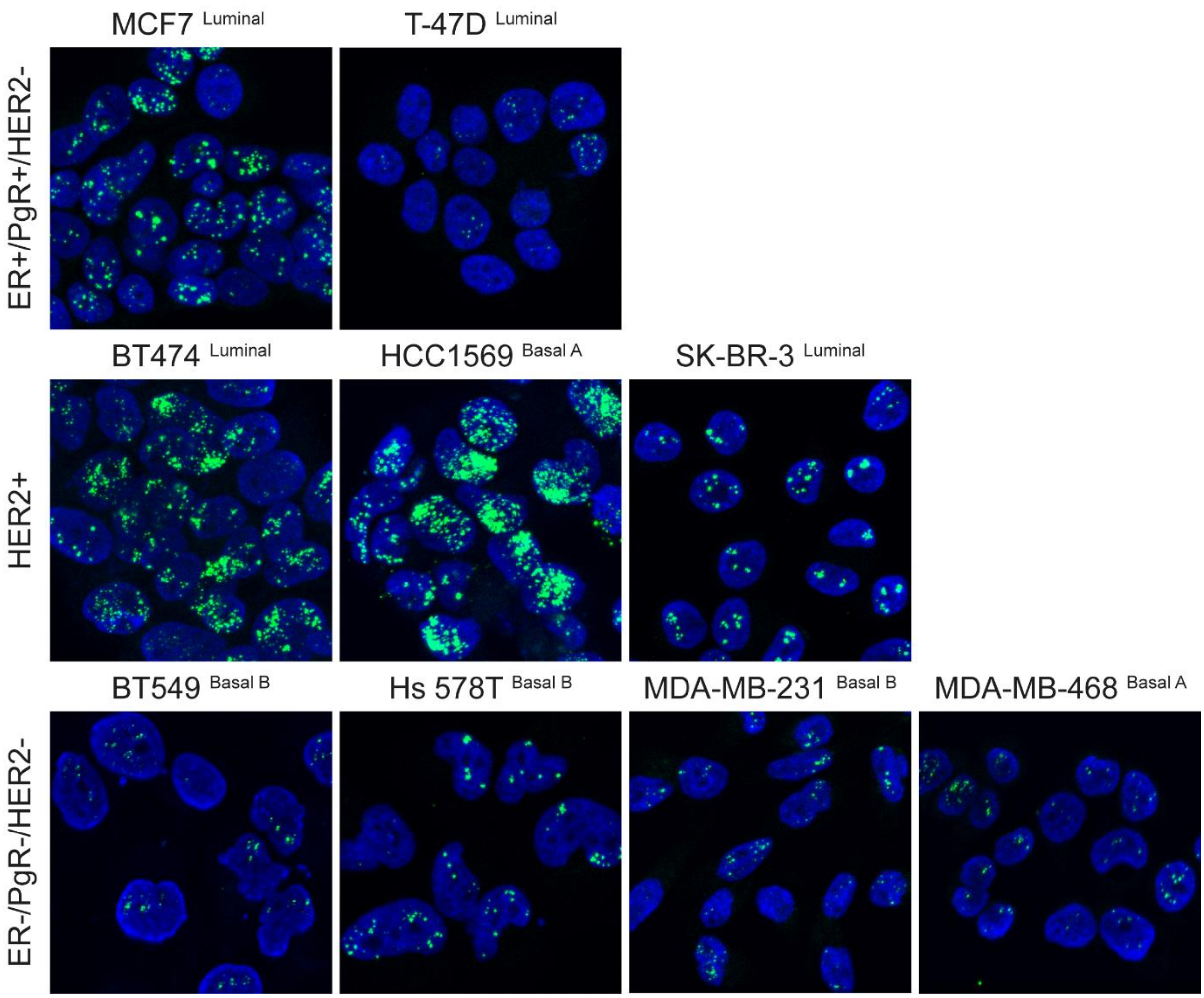

Supplementary figure 2: *NEAT1_2* expression and paraspeckle formation in a panel of nine breast cancer cell lines. Cells were subjected to RNA-fluorescent in situ hybridization (RNA-FISH) using probes recognizing the *NEAT1_2* isoform (green signal). DAPI (blue) was used to visualize the nuclei.

Supplementary Table 1. RT-qPCR primers.

Gene	Sequence
NEAT1_2	F: CGGAGGGTCTTGTAACACCAG
	R: AGTCCGGGCAACACAGAAAG
GAPDH	F: GAGCGAGATCCCTCCAAAAT
	R: AAATGAGCCCCAGCCTTCT
RPLP0	F: GCTGCTGCCCGTGCTGGTG
	R: TGGTGCCCCTGGAGATTTTAGTGG
B2M	F: TCATCCAGCAGAGAATGGAA
	R: TCTGAATGCTCCACTTTTTCAA

Forward (F), reverse (R).
